# Characteristics of Obstetric and Iatrogenic Urogenital Fistulas in Burkina Faso: A Cross-Sectional Study

**DOI:** 10.1155/2021/8838146

**Published:** 2021-01-20

**Authors:** Fasnéwindé Aristide Kabore, Stéphanie Dominique Amida Nama, Boureima Ouedraogo, Moussa Kabore, Adama Ouattara, Brahima Kirakoya, Gilles Karsenty

**Affiliations:** ^1^Department of Urology and Andrology, University Hospital Yalgado Ouedraogo of Ouagadougou, Ouagadougou, Burkina Faso; ^2^Urology Department, University Hospital of Tingandogo, Tingandogo, Burkina Faso; ^3^Urology Department, Souro Sanou University Hospital of Bobo-Dioulasso, Bobo-Dioulasso, Burkina Faso; ^4^Aix-Marseille Université, Marseille, France

## Abstract

**Objective:**

To compare the sociodemographic, clinical, and therapeutic characteristics of obstetric urogenital fistulas (OF) and iatrogenic urogenital fistulas (IF) treated in seven centers in Burkina Faso. *Material and Methods*. We carried out a cross-sectional study over a seven years' period (January 1, 2010 to December 31, 2016). We considered as iatrogenic all urogenital fistulas (UGF) occurred after elective caesarean section, gynecologic surgery (hysterectomy, myomectomy, and prolapse repair), or induced abortion. UGF following vaginal delivery after prolonged labor without obstetric maneuvers or caesarean section were considered as obstetric. UGF caused by other mechanisms (emergency caesarian section, congenital, and traumatic) were excluded from this study. The statistical analysis was carried out using version 14 of the STATA software. A logistic regression model was used to compare the two groups.

**Results:**

310 cases of UGF were included. IF accounted for 25.8% (*n* = 80) versus 74.2% (*n* = 230) for OF. The median age was 35 years for IF and 35.38 years for OF. The vesicovaginal fistulas were predominant (74.5%) in the two groups. All circumferential fistulas were found in the OF group. OF were frequently associated with residence in rural areas (OR = 1.8; CI = [1.05–3.1]), low level of education (OR = 5.4; CI = [2.3–12.9]), and a height under 158 cm (OR = 3.4 CI = [1.7–6.6]). Vaginal sclerosis was less common among IF (OR = 2.2; CI = [1–4.6]). The failure of surgical treatment after 3 months was more associated with OF (OR = 4.7; CI = [1.1–20.5]).

**Conclusion:**

OF were the most common, frequently affecting short women living in rural area and with low level of schooling. Fistulas were also more severe in the OF group. IF gave better results after surgical repair.

## 1. Introduction

Urogenital fistula (UGF) is an abnormal communication between the urinary and the genital tracts leading to involuntary loss of urine through the vagina. This society scourge mostly affects sub-Saharan Africa and South Asia regions with an incidence of 1.13 women in 1000 of childbearing age [1]. The social, economic, and psychological handicap caused by the UGF places them as a major public health problem [1–4]. The most common etiologies of UGF are obstetric (OF) and iatrogenic (IF). Previous studies in high-resource countries reported a majority of IF (83.2%) caused by pelvic surgery or radiation therapy [3–5]. On the other hand, OF concern the developing world resulting from obstructed labor and inadequate obstetric care. OF represent 87% to 95.6% of all UGF in low-resource countries [2, 3, 6]. Both OF and IF lead to an uncomfortable loss of urines but have different physiopathological mechanisms. Are their sociodemographic, clinical, and therapeutic aspects so different? There are studies on each of these two etiological types of UGF in Africa, but accounting for available data, there is a lack of comparative studies concerning the patterns of IF and OF. Most studies deal with factors associated with OF because of their predominance [2, 7]. However, according to Hilton [8], specialists of the field perceived a change of trend in developing countries, revealing an increasing proportion of IF. In Burkina Faso, OF's estimated prevalence is 23.1 per 100,000 births [9] and to our knowledge no study comparing OF and IF has been published. We hypothesized that IF differ from OF in both clinical and therapeutic characteristics. The present study aims to compare the sociodemographic, clinical, and therapeutic characteristics of IF and OF in Burkina Faso through a multicenter cross-sectional study. This will help to identify women at risk for each type of fistula and plan prevention strategies. It will also allow planning the surgery according to each type of fistula and predicting the results. Finally, it will draw the attention of health care providers to their responsibility in the prevention of IF.

## 2. Material and Methods

**Figure 1:**
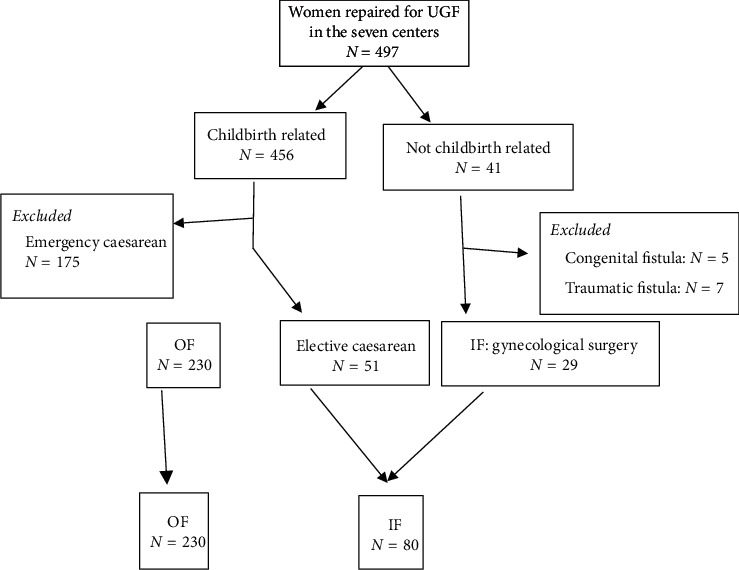
Flowchart of how we selected subjects for our study.

We carried out a cross-sectional study of hospital records on patients who had surgical repair for UGF in seven fistula treatment centers in Burkina Faso over a seven years' period (January 1, 2010 to December 31, 2016). The seven fistula treatment centers are University Hospital Yalgado Ouedraogo of Ouagadougou, Regional Hospital of Fada N'Gourma, Regional Hospital of Dori, Saint Camille Hospital in Ouagadougou, New Polyclinic of the Center in Ouagadougou, Medical Center with Surgical Antenna in Boromo, and Medical Center with Surgical Antenna of Schipphra in Ouagadougou. These seven centers are not the only urogenital fistulas treatment centers in Burkina Faso. Patients were followed up by a surgeon in each of the 7 centers. The survey was conducted by a single surgeon who collected data from the other surgeons in each of the 7 urogenital fistula centers. Two groups of UGF were individualized in this study: OF and IF. We considered as iatrogenic all UGF that occurred after prophylactic caesarean section, gynecologic surgery (hysterectomy, myomectomy, and prolapse repair), or induced abortion. UGF following vaginal delivery after prolonged labor without obstetric maneuvers or caesarean section were considered as obstetric [10]. We included patients with OF or IF and with a complete medical record. UGF caused by other mechanisms (emergency caesarian section, congenital, and traumatic) were excluded from this study.  Figure 1 shows the flowchart of how we selected subjects for the study.

The method of treatment of all fistulas was delayed repair after 3 months.

After approval of the local staff, data were collected on an individual and anonymous data sheet preserving patients' confidentiality at all centers. Information was collected on sociodemographic, obstetric, and previous surgeries history, fistula characteristics and history, intraoperative procedures, and surgical outcomes three months after discharge. The classification of UGF was performed through the Goh classification [11]. A dye test was performed whenever the patients reported for continuous urine leaking to determine the outcome of repair. Patients were followed for 3 months. Three surgical outcomes were considered: (i) failed or unclosed fistulas, (ii) closed fistula without stress urinary incontinence (SUI), and (iii) closed fistula with stress urinary incontinence.

Data entry and statistical analysis were performed using the software STATA 14 (StataCorp 2015, College Station, TX, USA). The dependent variable was the cause of the fistula. The modalities were the iatrogenic cause and the obstetric cause. Qualitative variables were presented in terms of numbers and percentages. Quantitative variables were presented as percentage (%), number (*n*), and average, with their standard deviation (SD), maximum (Max) and minimum (Min), or median values. Statistical analysis was performed to compare the two groups. Chi^2^ test or Fisher's exact test (where cell sizes were <5) were used to compare the frequencies. For the comparison of the averages in the two groups, we used the independent *t*-test. Odd Ratios (OR) with 95% confidence intervals (CI) were calculated from univariate tests to search for links between obstetric or iatrogenic causes and independent variables. In order to determine the significant differences between the two groups, we created a logistical regression model by including variables which have less than 10% missing data and with a disparity in the distribution. All *P* values less than 0.05 were considered statistically significant.

## 3. Results

During the study period, 497 cases of UGF were registered and 310 (62.4%) cases matched our inclusion criteria. The proportion of IF was 25.8% (*n* = 80) versus 74.2% (*n* = 230) for OF. The mean age of the patients at the time of diagnosis for the two groups was 37.2 ± 12.7 years. The mean age of IF and OF was, respectively, 37.8 ± 12.2 (min 19, max 70) and 36.9 ± 12.9 years (min 15, max 72). Most of these patients had no formal education (78.75% for IF and 95.6% for OF). The majority of patients in the OF group were from rural areas (58%) when the majority in IF group were from urban areas (58.75%). The mean duration of fistula was 65.8 ± 105.9 months and 116 ± 117.4 months, respectively, for IF and OF. The other sociodemographic characteristics and medical history of the patients are resumed in Table 1.

**Figure 2:**
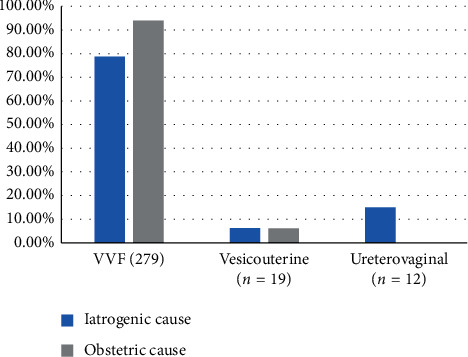
Type of UGF.

**Figure 3:**
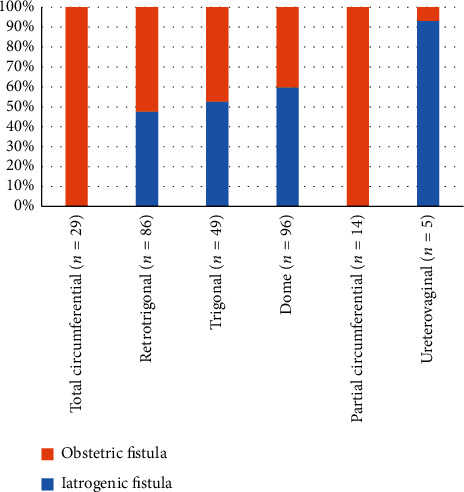
Seat of the vaginal fistula.

The majority of fistula was vesicovaginal in our two groups (Figure 2). Among these vesicovaginal fistulas (VVF), the most frequent location was supratrigonal (58%) in the two groups (Figure 3).

The mean size of the IF was 1.5 ± 1.3 cm (min: 0.2; max: 0.7) and 1.9 ± 1.6 cm (min: 0.2; max: 10) for the OF (*P* = 0.02). The perifistula sclerosis was most frequent in the OF group 23.9% (*n* = 55) versus 12.5% (*n* = 10) in IF group (*P* = 0.01). Using the Goh classification, 260 (83.9%) fistulas were classified as type 1-2 (Table 2).

All the OF followed obstructed labor in our study. Prophylactic caesarean section was the main etiology of IF in this study (63.8%, *n* = 51) (Table 3). The surgical procedures at the origin of IF have been performed by trained gynecologists in 53% (*n* = 42) of cases and by a general practitioner in 47% (*n* = 38).

Three months after surgery, the overall successful closure rate of the two groups was 79% (*n* = 245), failure of the repair represented 8.4% (*n* = 26), and residual stress urinary incontinence (RSUI) rate was 12.6% (*n* = 39). Failure of the repair occurred for 2.5% (*n* = 2) in the IF group and for 11% (*n* = 25) in OF (*P* = 0.02). The fistula was closed without SUI in 66 (82.5%) cases in the IF group and 178 (77.3%) cases in the OF group. Residual SUI represented 15% (*n* = 12) and 11.7% (*n* = 27) for IF and OF, respectively (*P* = 0.69). Logistic regression model is summarized in Table 4.

## 4. Discussion

Although during the last decade the number of IF treated in developing countries is increasing, the main etiology of UGF remains the obstetric cause [2, 3, 8, 12]. In our study, OF are three times more frequent than IF. Prolonged labor is the primary risk factor of OF. The current study with an average of labor duration over than two days (49.8 hours) confirms the results of previous reports from Africa [6,12–14]. The logistical difficulty to access to emergency obstetric care is a main trouble for women living in rural area. In our study, the obstetric cause was two times more common in rural areas than the iatrogenic cause (OR = 1.8, CI = [1.05–3.1]). According to rural residents interviewed in a study about OF in Burkina Faso, “it would be an endangered disease that was much more common at the time when there were no medical recourse” [15]. Traditional practices in rural populations and low medical literacy concerning the consequences of home births do not promote attendance at health facilities. The lack of medical literacy is a consequence of the low level of education in developing countries. In our study, patients with low level of schooling were four times more likely to be exposed to OF (OR = 5.4, CI = [2.3–12.9]). Tebeu et al. [13] reported an illiteracy rate ranging from 19 to 96% among patients with OF in sub-Saharan Africa.

In developing countries, the IF's frequency is increasing [8, 12, 16]. This is related to the increasing accessibility to surgery, especially caesarean section. Indeed, the World Health Organization (WHO) advocated for a caesarean section rate of 5 to 15% in developing countries to reduce the maternal mortality rate [17]. So, developing countries have initiated strategies to facilitate accessibility to caesarean section. But most of these caesarean sections often cause an additional risk to have an UGF because they are performed in ischemic tissues after a prolonged labor or when the fetus death in utero is recorded for several days [6, 14].

In Burkina Faso, the caesarean section's rate is 0.6% and a considerable number of UGF occurs after caesarean section [18]. In our series, elective caesarean was incriminated for 63.75% of IF. These results can be explained by the insufficiency of trained specialized medical doctors added to the lack of health infrastructures. In fact, concerning the IF, a gynecologist was the operator in only 42 cases (53%) in our study. Clinical officers and physician trained in basic surgery were involved in 38 cases (47%). Raassen et al. [6] reported 26.5% of IF cases for which a gynecologist was incriminated; the other UGF were caused by clinical officers and physician trained in basic surgery. In Burkina Faso, only 46% of caesarean sections are performed by a gynecologist, and amongst all caesareans performed, 12% are classified by experts as medically unjustified. Those are mostly carried out by nonspecialist agents [18]. We warn against these risky interventions because, as with any surgical procedure, caesareans are associated with short- and long-term risks for health. These risks are higher for women with limited access to appropriate obstetric care.

The most common type of UGF in our study was vesicovaginal (90%) according to the data reported in the medical literature [4, 6, 8, 16]. The high prevalence of VVF comes from the close adherence between the bladder and the vagina and the large contact area between its two organs. Depending on the etiology, the most affected anatomical area may vary. Studies report that, in IF, VVF are often high situated at the bladder dome, whereas in OF they are low situated with frequently cervical–urethral circumferential lesion [16]. Our results report an absence of correlation between the etiology of the UGF and the seat in the bladder wall except for circumferential lesions which are exclusively obstetric and ureteric lesions, which are exclusively iatrogenic. Onsrud et al. [16] also report that uterovaginal fistulas were more frequently iatrogenic. The diameter of the UGF is often related to the etiology. A large diameter is frequently described in OF [7, 19]. In our study, OF tended to be larger than IF (*P* = 0.02). The high propensity for OF to be large is related to their mechanism of occurrence. The size is determined by the ischemic zone, from which is removed the pressure ulcer giving way to the UGF. This ischemic area will cause tissue changes leading to fibrosis [14].

Logistic regression showed a high propensity for IF to be type 1 or 2 of the Goh classification. This testifies to the simplicity of most IF leading to the best successful fistula closure. Three months after the surgical repair, OF were almost five times more associated with failure of the repair of UGF in our study (*P* = 0.02). The predictive factors of treatment's failure are subject to controversies. Some authors reported only clinical and therapeutic implications while others pointed out the importance of the psychosocial status of the patient [7, 14, 20]. In our study, factors which can be regarded as predictive factors of failure are sclerosis, the loss of tissue, and the urethral destruction. These items were frequently associated with obstetric origin. That explained why the failure of the closure is more often seen in the obstetric group.

### 4.1. Limitations

One of the limitations of our study was the cases of fistula related to operative delivery for prolonged obstructed labor. It is difficult to tell if the fistula is due to surgery or prolonged ischemia. Raassen et al. reported the same difficulty [6]. For this reason, we excluded 175 cases of fistula from our study in order to have an homogeneous study population. However, this exclusion has considerably reduced the size of our population. Our data do not include all fistula treatment centers in Burkina Faso, so our results cannot be generalized to the entire population of Burkina Faso. Also because of the retrospective nature of this study, some data were missing.

## 5. Conclusion

Our study demonstrated that there are some important differences between IF and OF in Burkina Faso. OF mostly affected women living in rural areas, having a low level of education. OF give worst treatment's result than IF. IF are tiny, from type 1 or 2 of the Goh classification, less surrounded by sclerosis. IF are more frequent among women with at least a primary schooling level, living in urban areas. Although IF are looking less serious than OF, it is necessary to pay special attention to them because they lead to a loss of confidence from patients in health providers.

## Figures and Tables

**Table 1:** Sociodemographic, clinical, and therapeutic characteristics of patients.

	OF (*n*)	IF (*n*)	*P*	OR	IC = 95%
Patient's age at diagnosis	36.9 ± 12.9 years	37.8 ± 12.2 years	0.77	—	—
Area of residence					
Rural	124	29	0.04	1.8	1.05–3.1
Urban	99	42			
Missing: 16					

Educational level					
Less than primary school	183	63	<0.001	5.4	[2.3–12.9]
Higher than primary school	9	17			
Missing: 38					

Marital status before fistula					
Married	183	56	0.057	—	
Not married	0	2			
Missing: 69					

Height					
≤158 cm	103	45	<0.001	3.4	[1.7–6.6]
158 cm	20	30			
Missing: 112					

Duration of fistula					
>2 years	60	22	0.002	3.48	[1.5–7.6]
<2 years	18	233			
Missing: 187					

Circumferential fistula					
Yes	28	80	<0.001	—	
No	202	0			
Missing: 0					

Number of fistulas					
1	226	30	<0.001	0.25	[0.07–0.87]
>1	4	19			
Missing: 0					

Ureteral lesion					
Yes	0	73	<0.001	5.41	[1.5–19]
No	230	7			
Missing: 0					

Perifistula fibrosis					
Yes	55	10	0.01	2.2	[1–4.6]
No	175	70			
Missing: 0					

Urethral lesion					
Yes	48	73	0.004	3.2	[1.3–7.8]
No	182	6			
Missing: 0					

Fistula size	1.5 ± 1.3 cm				
Missing: 0		1.9 ± 1.6 cm	0.02	—	—

Labor duration	49.8 ± 32.5 h	N/A			
Surgical failure at 3 months					
Yes	25	2	0.02	4.7	[1.1–20.5]
No	205	78			
Missing: 0					

RSUI					
Yes	27	12	0.69	—	
No	178	66			
Missing: 27					

RSUI: residual stress urinary incontinence.

**Table 2:** The Goh classification of UGF.

	Iatrogenic fistula *N* = 80	Obstetric fistula *N* = 230
Goh's classification		
*T*1	38.75% (*n* = 31)	49.13% (*n* = 113)
*T*2	42.5% (*n* = 34)	35.65% (*n* = 82)
*T*3	18.75% (*n* = 15)	15.22% (*n* = 35)

**Table 3:** Etiologies of iatrogenic fistulas.

Etiologies	*n*	%
Prophylactic caesarean section	51	63.75
Hysterectomies	23	28.75
For uterine fibroids	19	23.75
For obstetric complications	3	3.75
For cervical neoplasia	1	1.25
Abortion	1	1.25
Prolapse repair	2	2.5
Uterine myomectomy	3	3.75

**Table 4:** Logistic regression model.

	OR	Std err	*z*	*P* > |*z*|	CI = 95%
Age (≤35 years/>35 years)	0.90	0.28	−0.34	0.732	[0.49–1.66]
Fistula size (≤1 cm/>1 cm)	0.35	0.15	−2.46	0.014	[0.15–0.8]
Sclerosis (yes/no)	1.16	0.55	0.32	0.752	[0.45–2.94]
Urethra lesion (yes/no)	0.26	0.18	−1.95	0.051	[0.07–1.008]
*T*1 of Goh's classification (yes/no)	0.078	0.06	−3.52	<0.001	[0.02–0.32]
*T*2 of Goh's classification (yes/no)	0.3	0.18	−2.01	0.045	[0.09–0.97]
Retrotrigonal fistula (yes/no)	1.8	1.07	1.00	0.318	[0.56–5.77]
Trigonal fistula (yes/no)	1.55	0.89	0.76	0.444	[0.50–4.79]
Bladder dome fistula (yes/no)	2.51	1.53	1.52	0.129	[0.76–8.27]
Area of residence (rural/urban)	0.45	0.15	−2.47	0.014	[0.24–0.85]
RSUI	0.51	0.3	−1.20	0.229	[0.17–1.53]

RSUI: residual stress urinary incontinence.

## Data Availability

The data supporting the findings of this study are available from the corresponding author upon request.
